# Reducing Objectification Could Tackle Stigma in the COVID-19 Pandemic: Evidence From China

**DOI:** 10.3389/fpsyg.2021.664422

**Published:** 2021-05-28

**Authors:** Youli Chen, Jiahui Jin, Xiangyang Zhang, Qi Zhang, Weizhen Dong, Chun Chen

**Affiliations:** ^1^Tongji Medical College, Wuhan Union Hospital, Huazhong University of Science and Technology, Wuhan, China; ^2^College of Humanities and Communication, Zhejiang University of Finance and Economics, Hangzhou, China; ^3^Department of Procurement, First Affiliated Hospital of Wenzhou Medical University, Wenzhou, China; ^4^School of Community and Environmental Health, College of Health Sciences, Old Dominion University, Norfolk, VA, United States; ^5^Department of Sociology and Legal Studies, University of Waterloo, Waterloo, ON, Canada; ^6^School of Public Health and Management, Wenzhou Medical University, Wenzhou, China

**Keywords:** stigma, COVID-19, self-categorization theory, core social motives, objectification, moderation analysis, China

## Abstract

Stigmatization associated with the coronavirus disease 2019 (COVID-19) is expected to be a complex issue and to extend into the later phases of the pandemic, which impairs social cohesion and relevant individuals' well-being. Identifying contributing factors and learning their roles in the stigmatization process may help tackle the problem. This study quantitatively assessed the severity of stigmatization against three different groups of people: people from major COVID-19 outbreak sites, those who had been quarantined, and healthcare workers; explored the factors associated with stigmatization within the frameworks of self-categorization theory and core social motives; and proposed solutions to resolve stigma. The cross-sectional online survey was carried out between April 21 and May 7, 2020, using a convenience sample, which yielded 1,388 valid responses. Employing data analysis methods like multivariate linear regression and moderation analysis, this study yields some main findings: (1) those from major COVID-19 outbreak sites received the highest level of stigma; (2) factors most closely associated with stigmatization, in descending order, are objectification and epidemic proximity in an autonomic aspect and fear of contracting COVID-19 in a controllable aspect; and (3) superordinate categorization is a buffering moderator in objectification–stigmatization relationship. These findings are important for further understanding COVID-19-related stigma, and they can be utilized to develop strategies to fight against relevant discrimination and bias. Specifically, reinforcing superordinate categorization by cultivating common in-group identity, such as volunteering and donating for containment of the pandemic, could reduce objectification and, thus, alleviate stigma.

## Introduction

Stigmatization of certain diseases makes many patients suffer both physically and mentally. In the case of coronavirus disease 2019 (COVID-19), however, even those without the illness are being stigmatized or dehumanized. It became a true side effect of the pandemic. Identifying factors contributing to stigmatizing behavior may facilitate efforts to prevent future stigmatization, improve mental health, and help the public to respect each other and fight against our true “enemy”: the virus.

COVID-19 has become a global pandemic on March 11, 2020 (WHO, 2020b). Although calls for the prevention of social stigma have gained attention, few quantitative studies on stigma during the COVID-19 pandemic have been done other than in the sphere of race and ethnicity (Devakumar et al., 2020; He et al., 2020). The paucity of literature in this field was exacerbated by the fact that most studies investigated people who had perceived discrimination toward themselves, instead of people who had stigmatized or discriminated against others (Cho et al., 2020).

This study hypothesize that in the context of COVID-19, social distancing and lockdown could result in autonomic and controllable aspects of stigmatization, accompanied with the incentive of self-protection. In order to reduce stigma, solutions should be developed to curb all aspects of stigmatizing behavior and cultivate a more inclusive and transparent social moral. The objectives of this study are, firstly, to assess the severity of stigmatization against certain groups of peoples; secondly, to identify factors associated with stigmatization; and lastly, to find effective solutions.

This study was carried out in China after Wuhan's lockdown has been lifted. It was a critical point in time in terms of ongoing efforts in the prevention and control of COVID-19 and the gradual normalization of daily lives. Since Wuhan, a city of 11 million, has reopened and all residents resumed their prelockdown movements, the new normal is anticipated to meet new challenges on multiple fronts, such as developing new measures for the epidemic's effective prevention, easing the public's remaining sense of uncertainty, fear, and anxiety. Meanwhile, stigma attachment became apparent to the city's residents, especially to those who would travel to other jurisdictions outside of the city.

Stigma is a complex topic. Understanding COVID-related stigma and fighting against discrimination can help the general public to truly restore prepandemic life. Otherwise, the impact of social discrimination could have more severe consequences than the pandemic itself. Although the survey was carried out in China, this study has implications for providing empirical evidence for understanding and addressing stigma in a global context.

### Conceptual Framework

A public health crisis causes the general public's fear and anxiety due to uncertainties. A diverse body of literature supports the idea that social exclusion during infectious disease epidemics operates as a behavioral immune system (Schaller and Park, 2011), which is seen as a rational process to proactively prevent oneself from exposure to the virus or pathogen. However, such fear and anxiety tends to flame hysteria and provoke irrational behavior like negative attitudes toward people, places, or things (NCIRD, 2020).

Stigma is defined as negative stereotyping. In the context of health, it is due to real or assumed negative association between a specific disease and a person or a group of people who share certain characteristics (Thornicroft et al., 2008), even though those being stigmatized against may not be posing any increased risk of pathogen transmission (Schaller and Neuberg, 2012). In the case of COVID-19, for example, people of certain ethnic backgrounds (e.g., Chinese or other Asians) (Chen et al., 2021), people from major epidemic outbreak sites, and anyone perceived to have been exposed to the virus, such as emergency responders or healthcare professionals, those who have recovered from COVID-19, and people who were under quarantine, may suffer from stigma.

During the COVID-19 pandemic, stigmatization against some groups of people has become a global issue, presenting as denial of services, social avoidance or rejection, verbal abuse, and even physical violence (imnepal.com, 2020). Racist assaults and attacks against Asians have been widely reported. Healthcare workers were asked to vacate their homes (Bagcchi, 2020). People from outbreak sites were being humiliated and shunned; some even had their personal information being leaked to the public. Many COVID-19 survivors were subject to discrimination at workplaces, healthcare settings, and their neighborhoods (Wang, 2020). Stigmatizing behaviors are causing psychological distress to those in certain groups. Such behavior also discourages people from seeking healthcare and being diagnosed. Moreover, it could undermine social cohesion and cause further social alienation, which will result in more severe problems in the society where all people should fight against the virus and not against each other.

Academic research on stereotyping and prejudice has focused more on the distinction between autonomic (implicit) and controlled (explicit) processes over the years. Automatic processes of prejudice involves the unconscious activation of well-learned set of associations, and it appears to be initiated by the presence of stimulus cues in the environment (Devine, 1989). In other words, prejudicial behavior and stigmatization may derive from implicitly held attitudes or stereotypes that the perceiver may be unaware and acted without intention. Thus, the automatic aspects (often categorization) of stigma are driven by social context.

Under the circumstance of a global pandemic, stigmatizing against marginalized groups is seemingly associated with perceived risks of transmitting the disease. A subtle manifestation of “objectification,” a form of dehumanization, for example, has become an all-too-familiar phenomenon in social media and everyday lives. The need for virus containment, control, and prevention measures and objective news reports are related to the neglect of the person's subjective experience in favor of objective, technologically mediated information, which has been described as “the denial of qualities associated with meaning, interest, and compassion” (Haslam, 2006).

Moreover, studies have shown that common conditions in daily life, such as multitasking (Stangor and Duan, 1991), distraction (Schneider et al., 2019), arousal, and stress (Wilder and Shapiro, 1989), add to cognitive load, all of which facilitate access to and application of one's stereotypes. During the coronavirus outbreak, the “infodemic” characterized by information explosion and overload has played a role in people's heightened worries, fears, anxieties, and depression, which affect people's rational judgment and decision-making ability. Some of them would turn to stigmatizing others as a type of accessible autonomic social-cognitive process to cope with their empathic distress. The normalization of certain expressions is likely to cause unconscious alienation of “other” in an intergroup context.

**Figure 1:**
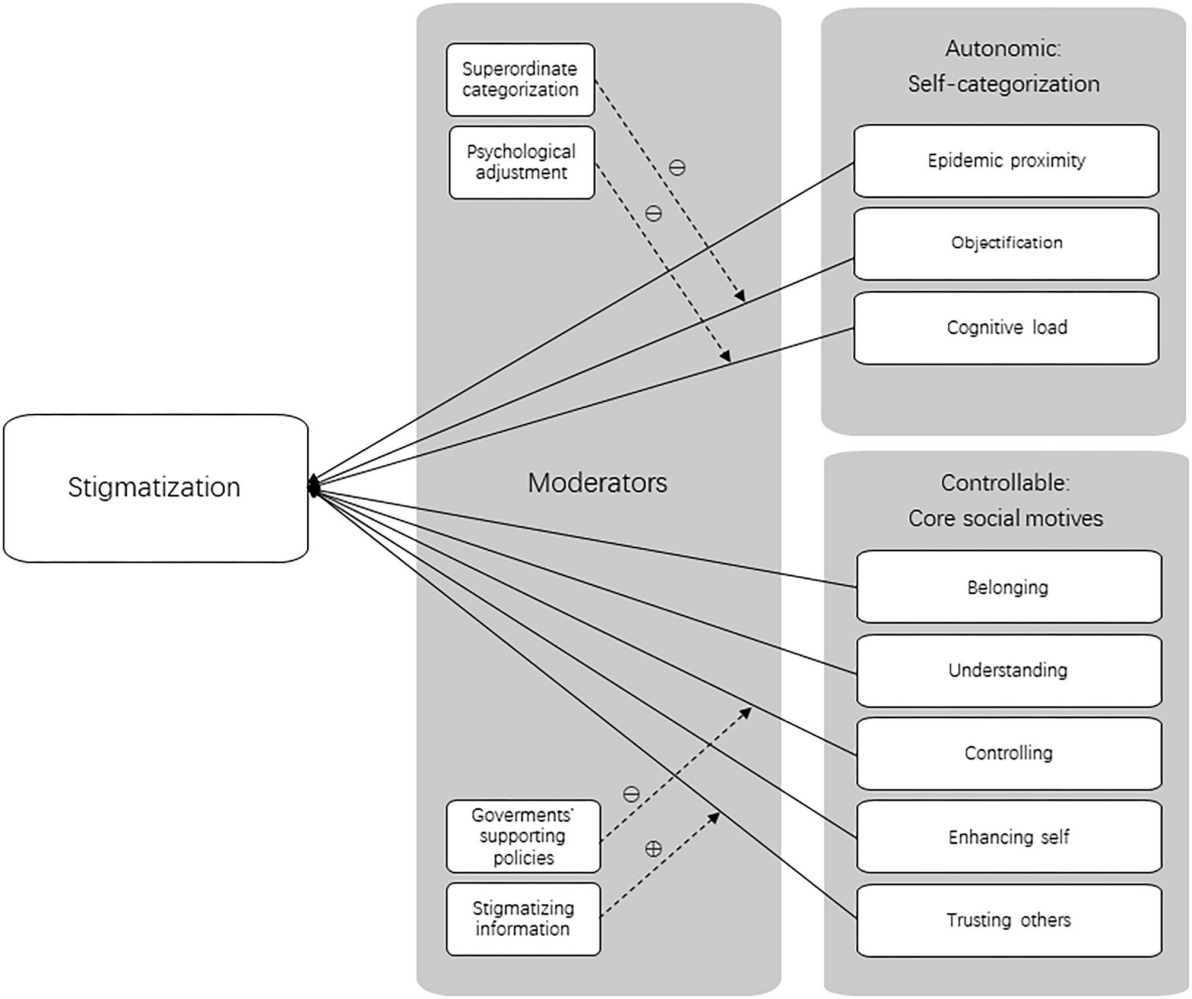
Theoretical models. Components in self-categorization theory and core social motives (BUC[K]ET) and proposed moderators.

Controllable aspects, however, are intentional and require active attention of the individual involved (Fiske, 1998). Controlling stigmatization depends largely on “motives,” which are fundamental psychological processes that drive people's thinking and behavior in certain social situations (Fiske, 2010). Five core social motives have been identified by personality and social psychologists over the years, known as BUC[K]ET: belonging (being part of a group), understanding (understanding what others in the groups believe), controlling (feeling of contingency between their actions and outcomes), self-enhancing (maintaining a degree of self-esteem), and trusting others (perceiving the social world as a benevolent place) (Fiske and North, 2013). Fulfilling these needs increases peoples' chances of social survival—humans' fundamental need as social beings. Aside from the obvious reason of being fearful of contracting the disease, one could stigmatize certain groups of people with the intention of staying in conformity with their own group and regaining a sense of control. These incentives are unrelated to health. Additionally, people highly in need of cognition and people self-consciously trying to be scientific operate in a more data-driven, less stereotypical mode (Aronson and Aronson, 2018). In the context of COVID-19, such cognitive styles along with sufficient information on the virus help people to understand the pandemic objectively and form their rational attitudes, mitigating fear and anxiety and reducing their tendency of stigmatization. According to Fiske et al. (Fiske, 2010), trusting people see the social world as largely benevolent, so they are more trustworthy, empathic, cooperative, and helpful. In contrast, failure to trust others can damage the effectiveness of our social organization (Rotter, 1967). The theoretical models based on this conceptual framework are presented in Figure 1.

### Hypotheses

Hypothesis 1: Autonomic processes (epidemic proximity, objectification, cognitive load) during the COVID-19 pandemic are associated with stigma toward people from outbreak hot spots, those who were under quarantine, and healthcare workers. Specifically, the lower level of epidemic proximity and the higher level of objectification and cognitive load, the higher likelihood that COVID-19-related groups would be stigmatized.

Hypothesis 2: Controllable processes (belonging, understanding, controlling, self-enhancing, and trusting others) during the COVID-19 pandemic are associated with stigmatizing those from pandemic outbreak sites like Wuhan, people who were discharged from quarantine sites, and healthcare workers. Specifically, the stronger these motives, the higher likelihood that perceivers would stigmatize those who are more closely associated with COVID-19.

Hypothesis 3: The relationship between factors in the framework of autonomic and controllable processes and stigma could be moderated by targeted solutions. Explicitly, we hypothesize that (1) superordinate categorization negatively moderates the relationship between objectification and stigmatization, (2) the sufficiency of psychological adjustment negatively moderates the relationship between cognitive load and stigmatization, (3) satisfaction with governments' supportive policies would have a buffering effect on the relationship between loss of control (financial threat, future hopelessness, and feeling of resource scarcity) and stigmatization, and (4) perception of stereotyping information enhances the association between the lack of interpersonal trust and stigmatization.

## Methods

### Ethics

The study was approved by the Ethics Committee of Wenzhou Medical University.

### Sampling and Participants

This study employed the non-random convenient sampling method. The respondents of this study voluntarily participated in a cross-sectional online survey, which was carried out between April 21 and May 7, 2020. Participants responded to a self-report questionnaire through an online crowdsourcing platform powered by www.wenjuan.com, which has functions equivalent to Google Forms. It was then attached to electronic messages and online posts on Chinese popular social media such as WeChat and QQ and disseminated through social networks. There were no incentives provided to those who took part in the survey. In total, 1,411 respondents filled out the questionnaire, and 1,388 of them were valid.

### Measures

This questionnaire was designed based on empirical evidence and available literature, to collect data to examine stigmatized people from major COVID-19 outbreak sites (e.g., Wuhan), people who were discharged from quarantine sites, and healthcare workers, and to explore its causes (Supplementary Table 1). Besides the respondents' demographic information, the questionnaire seeks to acquire data regarding (1) the level of stigmatization, (2) factors associated with stigmatization, and (3) possible solutions to contributing factors.

#### Demographics

Demographic questions in the survey include gender, age, residence, educational level, employment status, and marital status; they were chosen for their potential influence on discriminatory attitudes and behavior as identified in previous studies (5). To address the impact of the risk of virus transmission on avoidance behaviors, respondents were assigned scores 1–3 corresponding to low, medium, and high level of COVID-19 outbreak risk in their respective city of residence according to the information issued by the General Office of the State Council of the People's Republic of China (Xinhua News Agency, 2020).

#### Level of Stigmatization

The respondents' stigmatizing attitude was measured by assessing their desire for social distance from certain peoples, which was adopted from the Bogardus Social Distance Scale (Adewuya and Makanjuola, 2008). Questions include respondents' reluctance to share a room with people from a COVID-19 outbreak hot spot, people who were discharged from quarantine sites (recovered from COVID-19 after treatment in hospitals or quarantine sites or persons confirmed free of COVID-19 and discharged from quarantine sites), and healthcare workers (Q8–Q10). The three targeted groups were selected because they do not pose any public health risk, but were perceived as a health threat, according to reported daily life experiences and news reports. The scale ranged from “definitely not” (1) to “definitely yes” (5). Cronbach's α score of the stigmatization scale is 0.767, an acceptable level of reliability for exploratory work (Ursachi et al., 2015). The KMO value of the data is 0.644, and Bartlett's test was significant (χ^2^ = 1,189.582; *df* = 3; *p* < 0.001). Subsequently, an exploratory factor analysis was conducted, which included all three items accounting for 68.256% of the total variance, and factor loading and communality for each item are high (Supplementary Table 2). Since there was only one dimension, we did not carry out a confirmatory factor analysis.

#### Factors Associated With Stigma

Given the exploratory nature of this survey, attitudinal survey questions were formulated empirically and roughly fitted within the frameworks of self-categorization theory and BUC[K]ET to examine the factors contributing to stigma. This set of questions was selected from several existing scales, including NASA Task Load Index (NASA, 1988), the Need to Belong Scale (Leary et al., 2013), the Perceived Control Across Domains Scale (Davies, 2004), the Multidimensional Mortality Awareness Measure & Model (MMAMM) (Levasseur et al., 2015), the Interaction with Disabled Persons Scale (Gething and Wheeler, 2011), and the interpersonal trust scale (Rotter, 1967); next, they were reworded to fit into the COVID-19 pandemic context.

##### Autonomic Factors: Self-Categorization Theory

We used three questions shaped by the self-categorization theory: epidemic proximity (Q7, “Your role during the epidemic?”), objectification (Q11, “I think it is appropriate to refer to someone who returns from Hubei Province as ‘timebombs’.”), and cognitive load (Q13, “How frustrated or fatigued do/did you feel to cope with the pandemic?”).

The respondents' epidemic proximity was divided into three groups according to their roles' level of relevance and exposure to the pandemic. Those in direct contact with the epidemic, including healthcare workers (e.g., medical and nursing staff), epidemic control personnel (e.g., police, community service workers, volunteers), diagnosed COVID-19 patients, and suspected patients, were considered to have the most exposure to the epidemic and were assigned a score of 3. Family member of diagnosed patient(s), suspected patient(s), or health worker(s) and common people with a medical background (e.g., medical students) were those moderately exposed to the epidemic, and they were assigned a score of 2. The rest are deemed to have the least exposure to the epidemic, and they were assigned a score of 1.

The question addressing objectification was designed based on the objectifying language actually used for “controlling and preventing” the COVID-19 epidemic, while objectification underlies horizontal category displacement (Haslam, 2006), which reinforced categorization and stigmatization. We also set out to look for any purposeful and practical approaches to mitigate the relationship between objectification and stigmatization, such as “superordinate categorization” (Bavel et al., 2020), a process of reorganizing previously considered out-groups and in-groups into a single community with a common destiny. Thus, an additional question (Q12, “I'm willing to volunteer and/or donate money to help fight the pandemic.”) was designed to measure the intention for participation in the common course of fighting the pandemic, which predicts the salience of superordinate goals and the common in-group identity that leads to decategorization, superordinate categorization (Gaertner et al., 2000), and less stigmatization.

Studies show that excessive cognitive load takes up the cognitive resources (Wilder and Shapiro, 1989; Stangor and Duan, 1991; Bodenhausen, 2006; Schneider et al., 2019) needed to counteract autonomic processes, such as categorization. We modified a relevant question from the NASA Task Load Index (NASA, 1988) to determine participants' cognitive load (Q13, “How frustrated or fatigued do/did you feel while coping with the pandemic?”). Additionally, we examined the importance of a coping method of psychological adjustment (Q14, “Have you found any adjustment methods for the psychological impact brought to you by the pandemic?”).

##### Controllable Factors: BUC[K]ET

In this part of the questionnaire, two to three questions were used to tap into each of the five core social motives of BUC[K]ET, respectively. Need to belong [Q15, “Being apart from my family and friends due to the implementation of COVID-related restrictions (e.g., curfews, closures, social-distancing mandate, lockdown, etc.) for a long time does not bother me.”] and conformity (Q16, “People seldomly wore facial masks or respirators during early days of the outbreak. Did you wear a mask?”) demonstrated the motive of “belonging.” The motive of “understanding” consisted of knowledge-seeking efforts (Q17, “I made a great deal of efforts to learn about COVID-19.”) and knowledge acquisition (Q18, “Which modes of transmission are correct about COVID-19? [multiple-choice]”). The motive of “controlling” was measured by financial threat (Q19, “I'm worried that this pandemic is going to seriously affect my income and living standard.”), optimism (Q20, “I'm hopeful about the future.”), and feelings of resource scarcity (Q21, “Do you feel necessary to buy a lot of masks, medication, food and other household supplies in the following month?”). Worry (Q23, “I feel safe and do not worry about the risk of dying from contracting COVID-19.”), fearfulness (Q24, “When I think about the potential contraction of COVID-19, I feel nervous.”), and feelings of vulnerability (Q25, “Contact with someone who has high risk of getting COVID-19 reminds me of my own vulnerability.”) measured the motive of “enhancing self.” Lastly, the motive of “trusting others” was assessed by three questions measuring interpersonal trust (Q26, “Most public officials are honest about the current situation and sincere in their promises in their press briefings.”; Q27, “Most experts can be relied upon for telling the truth about COVID-19.”; Q28, “Most people can be relied upon to do their parts to control the pandemic.”). In addition, we examined participants' satisfaction with supportive policies implemented by the authorities (Q22) and their perception of stereotyping information from the media and others (Q29).

### Statistical Analysis

Q15, Q16, and Q23 were assigned scores reversely so that higher scores reflected greater corresponding attitudes. All the statistical analyses were performed using the Statistical Package for the Social Sciences (SPSS) 22.0 for Windows. Descriptive statistics were generated for all variables. Normality was tested using skewness and kurtosis of distribution (Kim, 2013). In the analysis, either an absolute skew value larger than 2 or an absolute kurtosis > 7 was used as reference values for determining substantial non-normality. All variables distributed normally except for location of residence (Supplementary Table 3).

Chi-square tests and one-way analysis of variance (ANOVA) were performed to assess differences in stigmatization among different demographic groups. Bonferroni's correction was applied for multiple comparisons. To examine different variables' closeness with stigma, a multiple linear regression analysis was conducted with attitudinal variables, while demographic variables were controlled.

Exploratory moderation analysis was performed to test the potential moderation effects of superordinate categorization, psychological adjustment, satisfaction with governments' measures, and stereotyping information between objectification, cognitive load, motive “controlling” (financial threat, optimism, and resource scarcity), and interpersonal trust, respectively, with stigmatization overall and against the three targeted groups, and moderation tests were conducted using the PROCESS Macro v2.13 in SPSS 22 (Hayes, 2012). In short, when variables were significant in the preceding multivariate analyses, we carried out separate, parallel, hierarchical regression analyses and examined the interaction plot.

## Results

### Descriptive Results

Table 1 presents the participants' major demographic characteristics. Of all the respondents (*n* = 1,388), 44.5% were males, and 90.3% of the respondents had received some postsecondary education and more. The median age of the respondents was 26–30 years old. Scores of the attitudinal variables are presented in Supplementary Table 5.

**Table 1:** Demographic characteristics.

**Demographics**	***N***	**Stigmatization**							
		**People from major outbreak sites**	**Bonferroni-corrected *p-*values**	**People discharged from quarantine sites**	**Bonferroni-corrected *p-*values**	**Healthcare workers**	**Bonferroni-corrected *p*-values**	**Overall**	**Bonferroni-corrected *p-*values**
Gender			<0.001^***^^a^		0.291^a^		0.345^a^		0.057^a^
Male	542 (44.5%)	3.44 ± 1.39		2.89 ± 1.40		1.98 ± 1.21		8.31 ± 3.32	
Female	828 (59.7%)	3.74 ± 1.30		3.05 ± 1.37		2.10 ± 1.20		8.90 ± 3.19	
Other	3 (0.2%)	1.67 ± 0.58		1.67 ± 0.58		1.67 ± 0.58		5.00 ± 1.73	
Age			0.504^b^		0.021^*^^b^		1^b^		0.378^b^
<18	14 (1.0%)	3.43 ± 1.83		2.71 ± 1.54		2.14 ± 1.41		8.29 ± 3.87	
18–25	651 (46.9%)	3.52 ± 1.25		2.83 ± 1.25		2.02 ± 1.10		8.37 ± 3.02	
26–30	220 (15.9%)	3.53 ± 1.43		2.96 ± 1.43		2.04 ± 1.23		8.54 ± 3.35	
31–40	215 (15.5%)	3.79 ± 1.37		3.19 ± 1.47		2.02 ± 1.29		9.00 ± 3.38	
41–50	209 (15.1%)	3.85 ± 1.37		3.21 ± 1.49		2.17 ± 1.32		9.22 ± 3.43	
51–60	47 (3.4%)	3.77 ± 1.60		3.28 ± 1.65		1.98 ± 1.33		9.02 ± 3.52	
>60	32 (2.3%)	3.53 ± 1.39		3.25 ± 1.41		2.31 ± 1.31		9.09 ± 3.76	
Place of residence			1^c^		1^c^		0.105^c^		0.783^c^
Risk level 1 cities	1,353 (97.5%)	3.62 ± 1.34		2.98 ± 1.38		2.04 ± 1.19		8.63 ± 3.23	
Risk level 2 cities	10 (0.7%)	3.80 ± 1.48		3.10 ± 1.60		2.20 ± 1.48		9.10 ± 4.18	
Risk level 3 cities	25 (1.8%)	3.76 ± 1.42		3.32 ± 1.57		2.84 ± 1.60		9.92 ± 3.73	
Educational level			1^b^		0.126^b^		0.063^b^		0.518^b^
Less than high school	115 (8.3%)	3.48 ± 1.54		3.10 ± 1.53		2.39 ± 1.41		8.97 ± 3.81	
High school, no diploma	19 (1.4%)	3.53 ± 1.54		2.84 ± 1.61		2.26 ± 1.41		8.63 ± 3.82	
High school diploma	54 (3.9%)	3.59 ± 1.56		2.93 ± 1.54		1.67 ± 1.01		8.19 ± 3.20	
Some college or some university, no diploma	557 (40.1%)	3.52 ± 1.24		2.81 ± 1.24		2.04 ± 1.12		8.37 ± 3.04	
College diploma	116 (8.4%)	3.69 ± 1.48		3.11 ± 1.54		2.12 ± 1.35		8.92 ± 3.64	
Bachelor degree	339 (24.4%)	3.77 ± 1.34		3.10 ± 1.42		1.94 ± 1.15		8.81 ± 3.19	
Post-graduate degree and higher	170 (12.2%)	3.76 ± 1.28		3.22 ± 1.40		2.14 ± 1.27		9.12 ± 3.23	
Employment status			<0.001^***^^a^		<0.001^***^^a^		0.147^a^		0.126^a^
Employed full-time	567 (40.9%)	3.74 ± 1.37		3.11 ± 1.45		1.99 ± 1.23		8.84 ± 3.30	
Employed part-time	17 (1.2%)	3.53 ± 1.46		2.82 ± 1.29		1.88 ± 0.99		8.24 ± 3.03	
Self-employed	99 (7.1%)	3.48 ± 1.53		3.00 ± 1.55		2.06 ± 1.33		8.55 ± 3.57	
Unemployed	49 (3.5%)	3.92 ± 1.41		3.29 ± 1.51		2.41 ± 1.41		9.61 ± 3.55	
Student	561 (40.4%)	3.49 ± 1.24		2.79 ± 1.24		2.04 ± 1.11		8.32 ± 3.04	
Retired	28 (2.0%)	3.75 ± 1.43		3.43 ± 1.48		2.39 ± 1.45		9.57 ± 3.99	
Unable to work	6 (0.4%)	3.00 ± 1.67		3.00 ± 1.67		2.17 ± 0.98		8.17 ± 2.99	
Marital status			<0.001^***^^a^		<0.001^***^^a^		0.012^a^		0.003^**^^a^
Married/living with a partner	583 (42.0%)	3.75 ± 1.41		3.16 ± 1.48		2.07 ± 1.29		8.99 ± 3.45	
Single (never married/widowed/divorced/separated)	695 (50.1%)	3.53 ± 1.26		2.84 ± 1.28		1.99 ± 1.10		8.36 ± 3.01	
Other	67 (4.8%)	3.34 ± 1.47		2.91 ± 1.33		2.27 ± 1.32		8.52 ± 3.62	
Total	1,388 (100%)	3.62 ± 1.344		2.99 ± 1.381		2.05 ± 1.203			

* *p < 0.05;*

** *p < 0.01;*

*** *p < 0.001*.

a *Chi-square test*.

b *One-way ANOVA*.

c *Kruskal–Wallis ANOVA*.

Participants showed varied levels of stigmatization toward people from the pandemic outbreak sites, people who were under quarantine, and healthcare workers, scoring 3.62 ± 1.344, 2.99 ± 1.381, and 2.05 ± 1.203, respectively (Table 1), which indicate that the highest level of stigmatization was reserved for those from COVID-19 epidemic hot spots (Supplementary Table 4).

Different demographic groups displayed different levels of stigmatization. Specifically, women, aged between 51 and 60, retirees, and those who are married are more likely to stigmatize certain population groups.

### Regression Results

As shown in Table 2, multivariate linear regression showed that those with a lower level of epidemic proximity, a tendency of objectification, and a higher level of fearfulness were associated with higher levels of overall stigmatization and against all targeted groups.

**Table 2:** Variables associated with stigmatization and discrimination.

**Variables**	**Multivariate regression^a^**							
	**People from major outbreak sites(s)**		**People discharged from quarantine sites**		**Healthcare workers**		**Overall**	
	***B*^b^**	***p*-values**	***B*^b^**	***p*-values**	***B*^b^**	***p*-values**	***B*^b^**	***p*-values**
Epidemic proximity	−0.119	0.014^*^	−0.209	<0.001^***^	−0.265	<0.001^***^	−0.593	<0.001^***^
Objectification	0.415	<0.001^***^	0.444	<0.001^***^	0.337	<0.001^***^	1.196	<0.001^***^
Cognitive load	0.030	0.346	0.009	0.785	−0.01	0.734	0.029	0.684
Need to belong	0.024	0.422	0.057	0.065	0.085	0.002^**^	0.165	0.013^*^
Conformity	−0.058	0.090	−0.023	0.519	0.054	0.083	−0.027	0.725
Knowledge-seeking efforts	0.049	0.231	−0.007	0.860	0.048	0.201	0.089	0.334
Knowledge acquisition	−0.024	0.62	0.009	0.861	0.003	0.935	−0.011	0.915
Financial threat	0.004	0.878	0.052	0.075	0.032	0.210	0.088	0.164
Optimism	0.004	0.929	0.033	0.46	−0.006	0.873	0.030	0.753
Feeling of resource scarcity	−0.021	0.520	0.031	0.361	0.095	0.001^**^	0.105	0.152
Worry	−0.050	0.257	0.045	0.327	0.046	0.248	0.041	0.681
Fearfulness	0.184	<0.001^***^	0.167	<0.001^***^	0.082	0.007^**^	0.433	<0.001^***^
Feeling of vulnerability	0.109	0.001^**^	0.032	0.336	0.007	0.810	0.147	0.04^*^
Trust in public officials	−0.068	0.034^*^	−0.030	0.369	0.004	0.883	−0.093	0.195
Trust in health experts	0.077	0.075	−0.019	0.667	−0.078	0.047^*^	−0.02	0.834
Trust in the general public	0.022	0.599	0.025	0.569	0.055	0.152	0.103	0.283

* *p < 0.05;*

** *p < 0.01;*

*** *p < 0.001*.

a *Multiple regression analysis controlling for gender, age, place of residence, educational level, employment status, and marital status*.

b *Unstandardized beta coefficient*.

Variables positively predicting overall stigmatization in order of effect were as follows: higher level of objectification (*B* = 1.196, *p* < 0.001), lower level of epidemic proximity (*B* = −0.593, *p* < 0.001), higher levels of fearfulness (*B* = 0.433, *p* < 0.001), and a stronger need to belong (*B* = 0.165, *p* = 0.013). Variables positively predicting stigmatization against people from the pandemic outbreak hot spots in order of effect are as follows: higher level of objectification (*B* = 0.415, *p* < 0.001), higher level of fearfulness (*B* = 0.184, *p* < 0.001), feeling of vulnerability (*B* = 0.109, *p* = 0.001), lower level of epidemic proximity (*B* = −0.119, *p* < 0.001), and less trust in public officials (*B* = −0.068, *p* = 0.034). Variables positively predicting stigmatization against people discharged from quarantine sites in order of effect are as follows: higher level of objectification (*B* = 0.444, *p* < 0.001), higher level of fearfulness (*B* = 0.167, *p* < 0.001), and lower level of epidemic proximity (*B* = −0.209, *p* < 0.001).

Higher levels of objectification (*B* = 0.337, *p* < 0.001), lower level of epidemic proximity (*B* = −0.265, *p* < 0.001), stronger feelings of resource scarcity (*B* = 0.095, *p* = 0.001), stronger need to belong (*B* = 0.085, *p* = 0.002), higher levels of fearfulness (*B* = 0.082, *p* = 0.007), and less trust in experts (*B* = 0.078, *p* = 0.047) are found to be associated with a higher likelihood of stigmatization toward healthcare workers.

### Moderating Effects

Scores of the proposed moderators are presented in Supplementary Table 5. Overall, participants reported relatively high superordinate categorization (4.28 ± 1.025), satisfaction with their governments' policies (3.91 ± 0.995), and adequate psychological adjustment (3.81 ± 1.061). Participants also reported a medium level of perception of stigmatizing information from the media and other individuals (3.12 ± 1.119).

Because the previous multivariate linear regression analysis suggested significant effects of objectification on stigmatization overall and that against all three targeted groups, feeling of resource scarcity on stigmatization against healthcare workers, trust in public officials on stigmatization against people from epidemic's outbreak hot spots, and trust in healthcare professionals on stigmatization against healthcare workers, exploratory moderation analyses were conducted to test the hypotheses that the superordinate categorization and satisfaction with governments' supportive policies negatively moderates the relationship between objectification, feeling of resource scarcity, and stigmatization, and that perception of stereotyping information enhances the association between the lack of interpersonal trust and stigmatization, using a two-step hierarchical multiple regression.

Specifically, we found a buffering role of superordinate categorization in the objectification–stigmatization relationship. In the first step, two variables were included: objectification and superordinate categorization. These variables accounted for a significant amount of variance in stigmatization [*R*^2^ = 0.206, *F*_(2,1,385)_ = 119.807, *p* < 0.001]. Next, the interaction term between objectification and superordinate categorization was added to the regression model, which accounted for a significant proportion of the variance in the level of stigmatization [Δ*R*^2^ = 0.004, Δ*F*_(1,1,384)_ = 6.400, *p* = 0.012, *B* = −0.0627, *t*(1,384) = −2.5298, *p* = 0.0115]. As superordinate categorization increased, the positive relationship between objectification and stigmatization decreased (Supplementary Figure 1). The effects of other imputed moderators did not exert significant moderating effects on the relationship between attitudinal variables and stigmatization, which confirmed Hypothesis 3 partially (Table 3).

**Table 3:** Moderation analysis.

**Model**	**Dependent variable**	**Independent variable**	**McFadden *R*^2^**	***p***	***B***	**SE**	***z***	***p***	**95% CI**
Model 1	Stigmatization against people from major outbreak sites	Constant	0.206	<0.001	3.62	0.03	112.56	<0.001	(3.56, 3.69)
		Objectification			0.47	0.03	18.6	<0.001	(0.42, 0.52)
		Superordinate categorization			0.09	0.03	2.74	0.0062	(0.02, 0.15)
		Objectification × superordinate categorization			−0.06	0.02	−2.53	0.0115	(−0.11, −0.01)
Model 2	Stigmatization against people discharged from quarantine sites	Constant	0.2171	<0.001	2.99	0.03	90.93	<0.001	(2.92, 3.05)
		Objectification			0.51	0.03	19.53	<0.001	(0.45, 0.56)
		Superordinate categorization			0.02	0.03	0.66	0.5113	(−0.04, 0.09)
		Objectification × superordinate categorization			−0.03	0.03	−1	0.3152	(−0.08, 0.02)
Model 3	Stigmatization against healthcare workers	Constant	0.168	<0.001	2.05	0.03	69.55	<0.001	(1.99, 2.11)
		Objectification			0.38	0.02	16.55	<0.001	(0.34, 0.43)
		Superordinate categorization			−0.06	0.03	−1.99	0.0473	(−0.12, 0)
		Objectification × superordinate categorization			−0.001	0.02	−0.05	0.9613	(−0.05, 0.04)
Model 4	Overall stigmatization	Constant	0.2842	<0.001	8.66	0.07	117.18	<0.001	(8.52, 8.81)
		Objectification			1.36	0.06	23.38	<0.001	(1.25, 1.48)
		Superordinate categorization			0.05	0.07	0.69	0.4876	(−0.09, 0.19)
		Objectification × superordinate categorization			−0.09	0.06	−1.57	0.1172	(−0.2, 0.02)
Model 5	Stigmatization against healthcare workers	Constant	0.06	<0.001	2.05	0.03	65.44	<0.001	(1.99, 2.11)
		Feeling of resource scarcity			0.24	0.03	8.64	<0.001	(0.19, 0.3)
		Satisfaction with governments' supportive policies			0.04	0.03	1.49	0.1362	(−0.01, 0.1)
		Feeling of resource scarcity × satisfaction with governments' supportive policies			0.05	0.02	1.94	0.0529	(0, 0.1)
Model 6	Stigmatization against people from major outbreak sites	Constant	0.0103	0.025	3.62	0.04	100.6	<0.001	(3.55, 3.69)
		Trust in public officials			0.03	0.03	1.13	0.2601	(−0.03, 0.1)
		Perception of stereotyping information			0.12	0.03	3.7	0.0002	(0.06, 0.18)
		Trust in public officials × perception of stereotyping information			−0.01	0.02	−0.49	0.6208	(−0.06, 0.04)
Model 7	Stigmatization against healthcare workers	Constant	0.0113	0.0013	2.05	0.03	63.76	<0.001	(1.99, 2.11)
		Trust in health experts			−0.05	0.04	−1.47	0.1425	(−0.12, 0.02)
		Perception of stereotyping information			0.11	0.03	3.74	0.0002	(0.05, 0.17)
		Trust in health experts × perception of stereotyping information			−0.004	0.03	−0.16	0.8722	(−0.06, 0.05)

## Discussion

The present work is the first attempt to analyze stigmatization in the COVID-19 pandemic in China. Superordinate categorization was found to be a buffering moderator on the relationship between objectification and discrimination. The following are the main findings of this study.

### Finding 1

This study shows that there are varied levels of stigmatization toward people with subtle connections to COVID-19. Those from major COVID-19 outbreak sites or hot spots received the highest level of stigmatization.

Our survey was conducted shortly after the end of Wuhan's lockdown, marking the transition of domestic pandemic control from a formerly static state to a dynamic state. This new normal increased people's sense of uncertainty and added fear and anxiety, which was indeed reflected in the survey results.

However, disease avoidance could be easily “simplified” to geographical discrimination: people in Hubei Province, especially in areas with concentrated outbreaks like Wuhan, need to be “treated differently.” It has been reported that “Wuhan” and “Hubei” people, vehicles, and even material goods have been segregated, blocked, and denied access, even after the epidemic situation in Hubei Province has been fundamentally improved, and most places became low-risk areas like those in the rest of the country. Beijing, Xinjiang, and Qingdao have also seen outbreaks since the new normal of pandemic prevention and control was marked by the lift of the Wuhan's lockdown, and local residents in these cities also faced similar discrimination.

Participants also reported a medium level of discrimination against those who were discharged from quarantine sites, disregarding reason for their quarantine. Experience from SARS (Lee et al., 2005) and Ebola (Mayrhuber et al., 2017) showed that such stigmatizing and avoidance behaviors against infectious disease survivors were felt at workplaces, hospitals, and neighborhoods, which fuels personal tragedies. Healthcare workers were also subject to discrimination, for instance, being barred from going back to their residential areas or using public transport and being publicly insulted. Social isolation and harassment can make an already challenging situation far more difficult for pandemic control as well as for healthcare providers (Bagcchi, 2020). In this study, however, the lowest level of stigmatization was found toward healthcare workers. It is likely that both accumulation of knowledge on COVID-19, for example, the modes of transmission of the virus, and the positive publicity about healthcare workers' fight against the pandemic and in saving lives despite their burnout, stress, and emotional burden helped them become a less stigmatized group. This is indicative of the importance of positive guidance of social values through mass media, making the general public aware of healthcare workers' sacrifices and contributions for safeguarding everyone's safety and health.

### Finding 2

Factors most closely associated with stigmatization within the frameworks of self-categorizing theory and core social motives, in descending order, are as follows: objectification, lower epidemic proximity, and fear of contracting COVID-19. The results partially confirmed Hypotheses 1 and 2.

Autonomic categorization and automatic associations to categories save cognitive resources, and they are the major culprits in the endurance of bias (Fiske, 1998). Three items in the questionnaire are about self-categorization: epidemic proximity, objectification, and cognitive load.

Firstly, objectification, one of the most powerful psychological processes behind prejudice, discrimination, and intergroup violence (Zimbardo et al., 2013), was found to be the most important contributing factor to stigmatization after the stage of achieving control of the COVID-19 virus in China. Referring to people coming from Hubei Province (a targeted group) as “timebombs” constitutes the denial of their mind, their complex inner life, and their basic humanity (Goffman, 1969); instead, it focuses only on their position as possible sources of infection. Specifically, objectification is characterized by horizontal category displacement and indifferent, instrumental, distancing attitudes and objectifying orientation toward them. Haslam et al. (Haslam, 2006) point out that objectification indexes the extent to which people see no-relatedness of the target group to others and express a lack of empathy for it, which leads to maltreatment and out-group alienation.

Secondly, higher levels of epidemic proximity are associated with less stigmatization. According to the contact hypothesis (Pettigrew and Tropp, 2008), increased exposure to out-groups reduces prejudice toward those groups. In the current social context, reducing stigmatization by increasing contact and exposure may operate through the challenge of creating cognitive dissonance (Aronson and Aronson, 2018), which occurs when people believe that those with some relationship to COVID-19 pose a health threat but cannot avoid direct or indirect contact with these people. In order to reduce a feeling of mental discomfort (dissonance) for having to live or work with them, they make alterations in their stigmatizing attitudes and behaviors. The general public with lower exposure to the disease, however, has less chance of adjusting their cognition.

Finally, “cognitive load” failed to show an influence on discriminatory tendencies in our multivariate analyses. However, the effect of cognitive overload on public perception and behaviors deserves further research regarding the “infodemic” nature of the COVID-19 pandemic.

Meanwhile, we used the BUC[K]ET framework to examine empirical factors.

First, fearfulness and feelings of vulnerability that attributed to the self-enhancing motive are the leading causes of stigmatization. Social exclusion during pandemics acts as a “behavioral immune system” against the disease-causing virus, since the virus is transmitted through personal contact. However, once people exhibit a predictable and situationally appropriate pattern of prejudice (e.g., disgust, disease-connoting cognitions, and behavioral avoidance) against individuals who truly do pose risk, they may exhibit the same behavioral pattern stigmatizing against other categories of people who pose no health risk at all, but merely appear morphologically similar or connected to actual patients in some superficial ways (Schaller and Neuberg, 2012). This finding can be accounted for with the combination of mortality salience, anxiety buffer, and death thought accessibility hypotheses of terror management theory (TMT). According to the TMT (Greenberg et al., 1997), humans need to manage their anxiety caused by “fear of death” in order to survive. During the COVID-19 outbreak, information highlighting negative aspects of the out-of-control epidemic, such as the living conditions of Wuhan residents and the overworked healthcare workers during the lockdown, elicited death-related thinking processes. Fear of death drives individuals to find ways to buffer fear, which include implanting meaning and value, such as cognitive consistency, stereotypes, social identity, etc. (Pyszczynski et al., 1999). Aversive responses such as stigmatization are likely to occur most strongly under conditions in which perceivers are (or merely perceive themselves to be) more vulnerable to pathogen infection (Schaller and Park, 2011).

Interestingly, in our survey, variables regarding self-enhancing motive showed less effect on the level of stigmatization than objectification. Categorization rather than self-preservation is the strongest promoter of discrimination, probably attesting to the irrationality of excessive avoidance of certain populations, especially in the later-stage COVID-19 pandemic when the stress of pathogen transmission has been reduced.

Second, however, in this study, responses implicating belonging, understanding, controlling, and trusting others' motives were found to affect stigmatization to a negligible level.

For one, belonging is a core social motive, and contact with and getting along with other people facilitates belonging (Fiske and North, 2013). In our survey, the level of the “need to belong” factor showed a slight positive correlation with stigmatization overall; it was directed against healthcare workers. It is possible that isolation and loneliness is taking a heavier toll on people who are more sociotropic and sensitive to solitary life. These individuals are also more likely to comply with the norms and expectations of others, if attempting a pleasant interaction (Chen et al., 1996), which entails compliance with various social norms, including stereotyping norms. Next, controlling motive was assessed with “feeling of financial threat,” “optimism,” and “feeling of resource scarcity.” In this study, a feeling of resource scarcity was found to be positively associated with stigmatization against healthcare workers. Anticipated as well as existing financial threats and resource scarcity have been cited frequently as factors contributing to feelings of threat and loss of personal control. Perceived control in multiple domains is important in an individual's life, enabling adaptive changes (Bandura, 1997); otherwise, an individual may become hopeless and helpless (Klein et al., 1976); under these circumstances, people tend to have an increased desire for control and the need to imbue control in other domains (Greenaway et al., 2014). Stereotyping is one such domain, because it works to provide a more structured and coherent reality for the person seeking control, even as it leads to stigmatization. Lastly, we found that less trust in public officials and health experts was associated with a higher level of stigmatization against people from the epidemic's outbreak hot spots and healthcare workers, separately. Results from our study stressed a correlational relationship between trust in authority figures representative of institutions and governments and discrimination, which supplements existing literature on leadership, trust, and compliance (Bavel et al., 2020). Trust acts as a mechanism for reducing complexity in social interactions and compensating for panic caused by insufficient information, producing a sense of security (Luhmann and Howard, 1979), which has significant social functions in the evolving pandemic.

### Finding 3

We found a significant buffering effect of superordinate goals on the objectification–stigmatization relationship—specifically, stigmatization against people from major outbreak sites, the most discriminated group according to survey results.

This could be explained by the common in-group identity model (Gaertner et al., 1993). At present, the intergroup behavior of people is so deeply affected by fear and anxiety over COVID-19 transmission that they have autonomously divided individuals into two groups: related and unrelated to the disease. Consequently, individuals from the “unrelated” group may discriminate against people from the “related” group.

However, epidemic control not only depends on the healthcare workers and other groups directly exposed to the epidemic, but also relies on activities such as relief donations and logistic support. Those who have the sense of social responsibility tend to pursue a superordinate goal—in this case, well-being of the human race instead of mere self-protection. The introduction and the salience of superordinate goals change peoples' conception of memberships from exclusive groups to more inclusive, superordinate groups, which creates and strengthens a common in-group identity (Gaertner et al., 2000). This process of decategorization and recategorization of perceived group boundaries reduces the salience of the original group boundaries and, consequently, ameliorates the original intergroup bias and conflict. In this light, we propose some practical solutions to the reality of pandemic-related stigmatization against certain members of society that could be useful for individuals, the media, and the government to implement. In order to buffer the effect of objectification on stigmatization, individuals, media, and the authorities should actively search for and share accurate information about the pandemic and the virus, using appropriate language, as the WHO advises (WHO, 2020a); engage with stigmatized groups in person or on social media to gain a proper perspective based on science instead of bias or fear; and support those who are in greater risk of contracting COVID-19, taking the initiative to participate in volunteer activities, make donations, and speak out against any uncivil and divisive behaviors.

Other proposed moderators, however, did not have significant effects in moderating the relationships between contributing factors and stigmatization, which leaves partially confirmed Hypothesis 3 and warrants further research.

### Limitations

While the study offers some novel and important findings, there are a number of limitations highlighting the need for caution in interpreting these findings.

First, researchers did not administrate multiple surveys over the course of the outbreak, yet public perception and behavior are expected to change over the course of an infectious disease outbreak. For example, targets of stigma were evolving, shifting from Hubei residents, to healthcare workers, and later, to returnees from abroad, migrant workers, and COVID-19 survivors. Correspondingly, updating policies could affect people's perception and behavior. However, we implemented the survey at a critical point of the progressing pandemic in China—immediately after Wuhan's lockdown was lifted—so that these findings could be a timely supplement for the effort to contain the outbreaks throughout its later courses in the pandemic, as well as provide a valuable reference for countries outside of China.

Second, the sample was composed of an online voluntary convenient online sample. Albeit its ability to quickly reach a relatively large and diverse sample, which fitted our intention to capture the characteristics of discrimination promptly after the lifting of Wuhan lockdown, the generalizability of this study is limited because of sample coverage and volunteer bias, which are common biases in online surveys. Specifically, young adults and well-educated persons were overrepresented, likely because of their relatively higher accessibility to the internet compared with their counterparts. Although normality of distribution was assessed and confirmed regarding participants' sociodemographic characteristics, caution in interpreting and generalizing these results is warranted.

Third, the survey questions were reworded from existing literature and developed through discussion among authors. Thus, the attitudinal questions that fit the frameworks of the self-categorization theory and BUC[K]ET are treated as independent variables and are not suitable for psychometric evaluation. Future studies can substantially advance our understanding by refining these measures, such as adding more survey questions in each attitudinal dimension.

Suggestions based on the results of this study can be utilized to fight stigmatization through the joint efforts of the government, media, and citizens by adjusting discrimination's autonomic aspects and controllable aspects. These findings also give rise to a whole new set of research questions that remain to be studied. Careful elucidation of these underlying mechanisms is an important direction for future research. In addition, cross-cultural studies on stigmatization during this coronavirus pandemic will yield meaningful findings.

## Data Availability Statement

The raw data supporting the conclusions of this article will be made available by the authors, without undue reservation.

## Ethics Statement

The studies involving human participants were reviewed and approved by the ethics committee of Wenzhou Medical University. Written informed consent to participate in this study was provided by the participants' legal guardian/next of kin.

## Author Contributions

YC: conceptualization, data curation, formal analysis, investigation, methodology, resources, software, and writing—original draft. JJ: conceptualization, investigation, resources, and writing—original draft. XZ: investigation and writing—review and editing. QZ: resources and writing—review and editing. WD: conceptualization, resources, and writing—review and editing. CC: conceptualization, funding acquisition, resources, supervision, and writing—review and editing. All authors contributed to the article and approved the submitted version.

## Conflict of Interest

The authors declare that the research was conducted in the absence of any commercial or financial relationships that could be construed as a potential conflict of interest.

## Supplementary Material

Supplementary Figure 1 Simple slopes of objectification predicting stigmatization for low average, and high superordinate categorization. The interaction plot showed a buffering effect that as superordinate categorization increased, the positive relationship between objectification and stigmatization decreased.

Supplementary Table 1 Questionnaire for investigation of factors associated with stigmatization after the lift of Wuhan lockdown in China.

Supplementary Table 2 Factor loadings from exploratory factor analysis.

Supplementary Table 3 Skewness and kurtosis.

Supplementary Table 4 Comparison of levels of stigmatization.

Supplementary Table 5 Summary of scores of variables.
