# Supplementary figures and images for: Reducing Objectification Could Tackle Stigma in the COVID-19 Pandemic: Evidence From China
Source: Front Psychol. 2021 May 28;12:664422. doi: 10.3389/fpsyg.2021.664422 (PMC8193049; doi:10.3389/fpsyg.2021.664422)

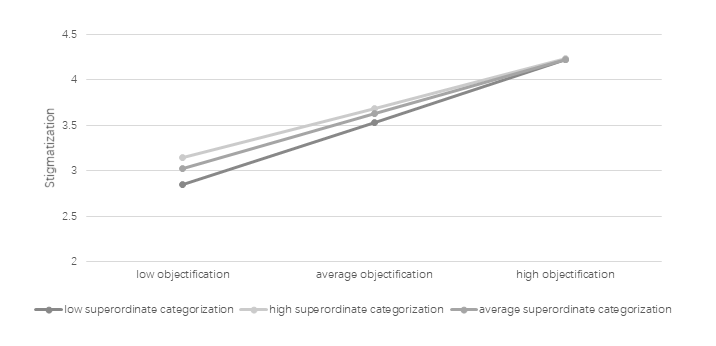

Supplement: Supplementary Figure 1 — Simple slopes of objectification predicting stigmatization for low average, and high superordinate categorization. The interaction plot showed a buffering effect that as superordinate categorization increased, the positive relationship between objectification and stigmatization decreased. [file Image_1.tif]
