# Supplementary material for: Reducing Objectification Could Tackle Stigma in the COVID-19 Pandemic: Evidence From China
Source: Front Psychol. 2021 May 28;12:664422. doi: 10.3389/fpsyg.2021.664422 (PMC8193049; doi:10.3389/fpsyg.2021.664422)
Supplement: Supplementary Table 1 — Questionnaire for investigation of factors associated with stigmatization after the lift of Wuhan lockdown in China. [file Table_1.docx]

Supplementary Table 1. Questionnaire for investigation of factors associated with stigmatization after the lift of Wuhan lockdown in China

| Items |  | Answer options |
| --- | --- | --- |
| Q1 | Gender: | O Male  O Female  O Other  O Prefer not to answer |
| Q2 | Age: | O 18 and younger  O 18-25  O 26-30  O 31-40  O 41-50  O 51-60  O 60 and older  O Prefer not to answer |
| Q3 | Location: (FILL IN THE BLANKS) | ________(City)________(Province) |
| Q4 | Education: | O Less than high school  O High school, no diploma  O High school diploma  O Some college, no diploma  O Some university, no diploma  O College diploma  O Bachelor degree and higher  O Prefer not to answer |
| Q5 | Employment: | O Employed full-time  O Employed part-time  O Self-employed  O Unemployed  O Student  O Retired  O Unable to work  O Prefer not to answer |
| Q6 | Marital status: | O Married/ Living with a partner  O Single (Never married /Widowed /Divorced /Separated)  O Other  O Prefer not to answer |
| Q7 | Your role during the epidemic: | O Healthcare worker (e.g. Medical staff, nursing staff, etc.)  O Epidemic control personnel (e.g. Police, community service worker, volunteer, etc.)  O Diagnosed patient  O Suspected patient  O Family member of diagnosed or suspected patient(s)  O Family member of medical worker(s)  O Common people with medical background (e.g. medical students, etc.)  O Common people |
| Q8 | Would you be upset if you have to share a room with someone from the COVID-19 outbreak sites (e.g., Wuhan)? | Definitely not 1 2 3 4 5 Definitely yes |
| Q9 | Would you be upset if you have to share a room with someone who were discharged from quarantines sites recently (recovered from COVID-19 after treatment in hospitals or quarantine sites, or confirmed free of COVID-19 and discharged from medical observation in quarantine sites)? | Definitely not 1 2 3 4 5 Definitely yes |
| Q10 | Would you be upset if you have to share a room with a healthcare worker during the pandemic? | Definitely not 1 2 3 4 5 Definitely yes |
| Q11 | I think it is appropriate to refer to someone who return from Hubei Province as “timebombs”. | Definitely not 1 2 3 4 5 Definitely yes |
| Q12 | I’m willing to volunteer and/or donate money to help fight the pandemic. | Definitely not 1 2 3 4 5 Definitely yes |
| Q13 | How frustrated or fatigued do/did you feel while coping with the pandemic? | Very low 1 2 3 4 5 Very high |
| Q14 | Have you found any adjustment methods for the psychological impact brought to you by the pandemic? | Definitely not 1 2 3 4 5 Definitely yes |
| Q15 | Being apart from my family and friends due to the implementation of COVID-related restrictions (e.g., curfews, closures, social-distancing mandate, lockdown, etc.) for a long time does not bother me. | Strongly disagree 1 2 3 4 5 Strongly agree |
| Q16 | People seldomly wore facial masks or respirators during early days of the outbreak. Did you wear a mask? | Definitely not 1 2 3 4 5 Definitely yes |
| Q17 | I made a great deal of efforts to learn about COVID-19. | Strongly disagree 1 2 3 4 5 Strongly agree |
| Q18 | Which modes of transmission are correct about COVID-19? [multiple-choice] | O Airborne  O Transmitted via droplets (e.g. sneeze)  O Transmitted via close contact |
| Q19 | I'm worried that this pandemic is going to seriously affect my income and living standard. | Strongly disagree 1 2 3 4 5 Strongly agree |
| Q20 | I’m hopeful about the future. | Strongly disagree 1 2 3 4 5 Strongly agree |
| Q21 | Do you feel necessary to buy a lot of masks, medication, food and other household supplies in the following month? | Definitely not 1 2 3 4 5 Definitely yes |
| Q22 | I’m satisfied with governments’ measures that support peoples whose livelihoods are affected by the COVID-19. | Strongly disagree 1 2 3 4 5 Strongly agree |
| Q23 | I feel safe and do not worry about the risk of dying from contracting COVID-19. | Strongly disagree 1 2 3 4 5 Strongly agree |
| Q24 | When I think about the potential contraction of COVID-19, I feel nervous. | Strongly disagree 1 2 3 4 5 Strongly agree |
| Q25 | Contact with someone who has high risk of getting COVID-19 reminds me of my own vulnerability. | Strongly disagree 1 2 3 4 5 Strongly agree |
| Q26 | Most public officials are honest about the current situation and sincere in their promises in their press briefings. | Strongly disagree 1 2 3 4 5 Strongly agree |
| Q27 | Most experts can be relied upon for telling the truth about COVID-19. | Strongly disagree 1 2 3 4 5 Strongly agree |
| Q28 | Most people can be relied upon to do their parts to control the pandemic. | Strongly disagree 1 2 3 4 5 Strongly agree |
| Q29 | The COVID-19-related information from the media and other individuals reinforces stereotypes. | Strongly disagree 1 2 3 4 5 Strongly agree |
