# Supplementary material for: Reducing Objectification Could Tackle Stigma in the COVID-19 Pandemic: Evidence From China
Source: Front Psychol. 2021 May 28;12:664422. doi: 10.3389/fpsyg.2021.664422 (PMC8193049; doi:10.3389/fpsyg.2021.664422)
Supplement: Supplementary Table 2 — Factor loadings from exploratory factor analysis. [file Table_2.DOCX]

Supplementary Table 2. Factor loadings from exploratory factor analysis

|  | **Factor** | **Communalities** |
| --- | --- | --- |
|  | 1 |  |
| **Stigmatization against people from major COVID-19 outbreak sites** | 0.825 | 0.680 |
| **Stigmatization against people discharged from quarantine sites** | 0.888 | 0.788 |
| **Stigmatization against healthcare workers** | 0.762 | 0.580 |
| **Explained variance (%)** | 68.256 |  |
