# Supplementary material for: Reducing Objectification Could Tackle Stigma in the COVID-19 Pandemic: Evidence From China
Source: Front Psychol. 2021 May 28;12:664422. doi: 10.3389/fpsyg.2021.664422 (PMC8193049; doi:10.3389/fpsyg.2021.664422)
Supplement: Supplementary Table 3 — Skewness and kurtosis. [file Table_3.DOCX]

Supplementary Table 3. Skewness and kurtosis

| **Variables** | **N** | **Skewness** | **Kurtosis** |
| --- | --- | --- | --- |
| Gender | 1373 | -0.386 | -1.708 |
| Age | 1388 | 0.859 | -0.246 |
| Place of residence | 1388 | 6.566 | 42.264 |
| Educational level | 1370 | -0.529 | -0.072 |
| Employment status | 1327 | -0.032 | -1.806 |
| Marital status | 1345 | 0.306 | -0.729 |
| Social identity | 1388 | 1.057 | -0.154 |
| Stigma (people from major COVID-19 outbreak sites) | 1388 | -0.499 | -1.003 |
| Stigma (people discharged from quarantine sites) | 1388 | 0.092 | -1.196 |
| Stigma (healthcare workers) | 1388 | 1.016 | 0.151 |
| Overall stigma | 1388 | 0.111 | -0.764 |
| Objectification | 1388 | 0.705 | -0.544 |
| Cognitive load | 1388 | 0.372 | -0.603 |
| Need to belong | 1388 | 0.203 | -0.743 |
| Conformity | 1388 | 1.867 | 2.641 |
| Knowledge-seeking efforts | 1388 | -0.846 | 0.301 |
| Knowledge acquisition | 1388 | -1.567 | 1.754 |
| Financial threat | 1388 | -0.370 | -0.816 |
| Optimism | 1388 | -1.203 | 1.039 |
| Feeling of resource scarcity | 1388 | 0.335 | -0.542 |
| Worry | 1388 | 0.915 | 0.581 |
| Fearfulness | 1388 | 0.131 | -0.724 |
| Feeling of vulnerability | 1388 | -0.725 | -0.284 |
| Trust in public officials | 1388 | -0.707 | -0.423 |
| Trust in health experts | 1388 | -0.538 | 0.169 |
| Trust in the general public | 1388 | -0.536 | -1.708 |
| Superordinate categorization | 1388 | -1.427 | 1.463 |
| Psychological adjustment | 1388 | -0.665 | 0.032 |
| Satisfaction of governments’ measures | 1388 | -0.533 | -0.379 |
| Stigmatizing information | 1388 | -0.118 | -0.415 |
