# Supplementary material for: Reducing Objectification Could Tackle Stigma in the COVID-19 Pandemic: Evidence From China
Source: Front Psychol. 2021 May 28;12:664422. doi: 10.3389/fpsyg.2021.664422 (PMC8193049; doi:10.3389/fpsyg.2021.664422)
Supplement: Supplementary Table 4 — Comparison of levels of stigmatization. [file Table_4.docx]

Supplementary Table 4. Comparison of levels of stigmatization

| Comparisons | mean±SD | p-values |
| --- | --- | --- |
| People from major COVID-19 outbreak sites – People discharged from quarantine sites | 1.164 ± 0.031 | <0.001 |
| People from major COVID-19 outbreak sites – Healthcare workers | 1.408 ± 0.038 | <0.001 |
| People discharged from quarantine sites – Healthcare workers | 1.258 ± 0.034 | <0.001 |
