# Supplementary material for: Reducing Objectification Could Tackle Stigma in the COVID-19 Pandemic: Evidence From China
Source: Front Psychol. 2021 May 28;12:664422. doi: 10.3389/fpsyg.2021.664422 (PMC8193049; doi:10.3389/fpsyg.2021.664422)
Supplement: Supplementary Table 5 — Summary of scores of variables. [file Table_5.DOCX]

Supplementary Table 5. Summary of scores of variables

| **Variables** | **N** | **Score (mean ± SD)** |
| --- | --- | --- |
| Epidemic proximity | 1388 | 1.49 ± 0.682 |
| Objectification | 1388 | 2.28 ± 1.277 |
| Cognitive load | 1388 | 2.56 ± 1.186 |
| Need to belong | 1388 | 2.78 ± 1.212 |
| Conformity | 1388 | 1.59 ± 1.054 |
| Knowledge-seeking efforts | 1388 | 4.10 ± 0.934 |
| Knowledge acquisition | 1388 | 3.55 ± 0.728 |
| Financial threat | 1388 | 3.38 ± 1.265 |
| Optimism | 1388 | 4.25 ± 0.941 |
| Feeling of resource scarcity | 1388 | 2.58 ± 1.142 |
| Worry | 1388 | 1.84 ± 0.882 |
| Fearfulness | 1388 | 2.92 ± 1.194 |
| Feeling of vulnerability | 1388 | 3.83 ± 1.157 |
| Trust in public officials | 1388 | 3.86 ± 1.166 |
| Trust in health experts | 1388 | 3.88 ± 0.900 |
| Trust in the general public | 1388 | 3.89 ± 0.977 |
| Superordinate categorization | 1388 | 4.28 ± 1.025 |
| Psychological adjustment | 1388 | 3.91 ± 0.995 |
| Satisfaction of governments’ measures | 1388 | 3.81 ± 1.061 |
| Stigmatizing information | 1388 | 3.12 ± 1.119 |
